# miComplete: weighted quality evaluation of assembled microbial genomes

**DOI:** 10.1093/bioinformatics/btz664

**Published:** 2019-08-22

**Authors:** Eric Hugoson, Wai Tin Lam, Lionel Guy

**Affiliations:** Department of Medical Biochemistry and Microbiology, Science for Life Laboratories, Uppsala University, Uppsala SE-751 23, Sweden; Department of Microbial Population Biology, Max Planck Institute for Evolutionary Biology, Plön D-24306, Germany; Department of Microbial Population Biology, Max Planck Institute for Evolutionary Biology, Plön D-24306, Germany; Department of Medical Biochemistry and Microbiology, Science for Life Laboratories, Uppsala University, Uppsala SE-751 23, Sweden

## Abstract

**Summary:**

Metagenomics and single-cell genomics have revolutionized the study of microorganisms, increasing our knowledge of microbial genomic diversity by orders of magnitude. A major issue pertaining to metagenome-assembled genomes (MAGs) and single-cell amplified genomes (SAGs) is to estimate their completeness and redundancy. Most approaches rely on counting conserved gene markers. In miComplete, we introduce a weighting strategy, where we normalize the presence/absence of markers by their median distance to the next marker in a set of complete reference genomes. This approach alleviates biases introduced by the presence/absence of shorter DNA pieces containing many markers, e.g. ribosomal protein operons.

**Availability and implementation:**

miComplete is written in Python 3 and released under GPLv3. Source code and documentation are available at https://bitbucket.org/evolegiolab/micomplete.

**Supplementary information:**

Supplementary data are available at *Bioinformatics* online.

## 1 Introduction

The developments of high-throughput sequencing have led to an ever-increasing affordability and availability of large-scale sequencing projects. Vast amounts of metagenomic data is generated, leading to the publication of thousands of metagenome-assembled genomes (MAGs) from uncultured microorganisms (e.g. Parks *et al.*, 2017). Genomes from uncultured microorganisms may also be obtained by sorting cells on a flow cytometer, amplifying and sequencing their DNA. The resulting single-cell amplified genomes (SAGs) have also contributed to widely increase our knowledge of microbial diversity (e.g. Rinke *et al.*, 2013).

Assessing the quality of MAGs and SAGs has become paramount. Metagenomic data is difficult to correctly assemble and bin into MAGs, with most MAGs missing contigs and/or being contaminated with foreign contigs. As for SAGs, their completeness varies widely, with virtually no SAG being 100% complete. Most methods estimate level of completeness and contamination (or redundancy) of SAGs and MAGs by identifying single copy, conserved marker genes. The fraction of identified markers corresponds to genome completeness, while additional copies represent either contamination or redundancy (Rinke *et al.*, 2013). This approach is implemented e.g. in CheckM (Parks *et al.*, 2015) and BUSCO (Simão *et al.*, 2015).

So far, all markers are considered as equally contributing to completeness or redundancy. However, markers are not uniformly distributed around prokaryotic chromosomes, and a certain amount of linkage is conserved even across long evolutionary distances (e.g. Rogozin *et al.*, 2002; Lathe *et al*., 2000). This is especially important since commonly used marker sets often include ribosomal protein genes, which are organized in conserved operons: thus, the presence or absence of ribosomal protein genes (or of other markers generally close to others) should contribute to completeness and redundancy less than that of other non-clustered genes.

## 2 Materials and methods

In miComplete, we implement a method to reduce potential bias introduced by the presence or absence of genetically linked markers. The selected set of markers is first identified in a set of representative, complete, closed chromosomes. For each marker in each genome, half the distance to the next marker upstream and downstream is recorded, and the sum of the two is normalized to the genome size. The median over all genomes is then used as weight when calculating the completeness or redundancy for query genomes. In a set of 105 highly conserved bacterial markers, the weights, inferred from a set of 1 175 genomes representative of all bacteria (see below), ranged over three orders of magnitude, from 1.02e-5 (Ribosomal protein L22) to 3.17e-2 (RecR). The distribution of weights for this particular set is shown in Supplementary Figure S1. The square root of the sum over the weights’ standard deviations is used as a measure of the uncertainty attached to a particular set of weights.

## 3 Implementation

miComplete is written in Python 3, and relies, among others, on Numpy and Biopython (Cock *et al.*, 2009). To identify completeness and redundancy, miComplete aligns a set of Hidden Markov Models (HMM) derived from single copy markers to the genomes of interest. First, prodigal (Hyatt *et al.*, 2010) is used to identify protein-coding genes in unannotated genomes. Second, HMMER3 (Mistry *et al.*, 2013) identifies hits to the set of HMM among the annotated genomes, employing both a simple e-value cutoff for initial matching as well as a combination of bias- and best single domain score to evaluate the quality of matches. HMM sets can be either provided by the user or selected from the (currently) two prebuilt sets, one for bacteria and one for archaea, derived from previous work (Guy, 2017; Rinke *et al.*, 2013). Completeness is the fraction of markers identified and redundancy the fraction of additional copies of markers.

miComplete calculates the weights of a marker set in a set of complete genomes, using then these weights to infer weighted completeness and redundancy in a query set of MAGs or SAGs. Alternatively, the user may use the precalculated weights associated with the inbuilt marker sets. Results are output in a tabulated list format, including the estimated weighted completeness and redundancy and a list of assembly statistics (genome length, GC-content, N- and L50, etc.). The estimated error attached to the weights and other metadata are also part of the output.

## 4 Benchmarking

To estimate the effectiveness of the new weighted approach, we simulated a set of MAGs from complete genomes into contigs, with varying completeness and redundancy. To obtain realistic contig lengths, a distribution of contig lengths was built from 2631 draft MAGs (Tully *et al.*, 2018) recovered from the Tara Oceans dataset (Karsenti *et al.*, 2011). The resulting distribution was used to randomly sample contig lengths to split a genome into. Contigs were then randomly removed to meet desired completeness level. To simulate redundancy, all other contigs were pooled and randomly added to each artificial MAG up to the desired level. The code used to calculate the contig length distribution and to simulate MAGs is freely available from https://bitbucket.org/EricHugo/randmag.

**Fig. 1.**
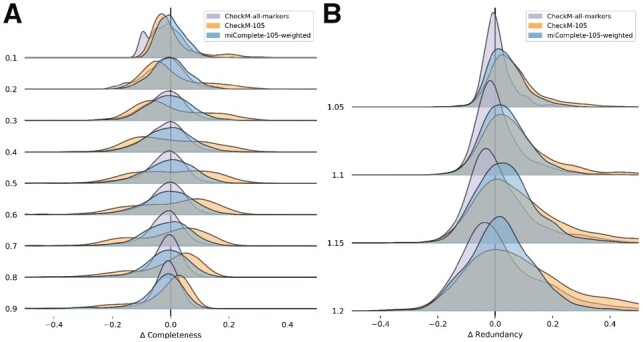
Ridgeplots of errors in estimating (**A**) completeness and (**B**) redundancy with CheckM, using a fixed marker set (orange) or default settings (purple) and miComplete (blue). Each stapled plot represents the distribution of error (difference between observed and expected value) for increments of simulated completeness (**A**) or redundancy (**B**)

Levels of completeness were simulated (0.1–0.9, increment 0.1) using this method, along with levels of redundancy (1.05–1.2, increment 0.05). For each increment, 10 000 MAGs were simulated from 1175 complete, representative genomes. These genomes were chosen with phyloSkeleton (Guy, 2017) to select one genome from each genus across all bacteria containing at least one complete genome (Supplementary Table S1). Results from miComplete and CheckM were then compared, employing the same set of markers on the same artificial MAGs. CheckM was run with the resource-intensive default settings for a subset of 1000 MAGs (Fig. 1).

Across all levels of completeness tested, the weighting strategy of miComplete yields an observed median completeness closer to the expected value than without weighting, when using the same set of markers (Fig. 1A). The distribution of estimated completeness also showed a lower variance with the weighting strategy than without. Redundancy estimations showed a similarly reduced variance with the weighting strategy, although the median is similarly close to the expected value for both strategies, only slightly favouring the weighted approach (Fig. 1B). Running the largest dataset of the estimations (1.2 redundancy) from the set above required an average of 9.81 s per simulated MAG for CheckM (fixed marker set) and 2.72 s for miComplete, on the same node using 12 threads for execution. It should be noted that CheckM contains a very large range of other features, among others automatically selecting markers, explaining its higher runtime and its generally better results with default settings.

In summary, miComplete allows a rapid and robust quality evaluation of MAGs and SAGs. Through weighting markers according to their conserved genetic linkage yields completeness and redundancy estimations that are less biased and have narrower distributions.

## Supplementary Material

btz664_Supplementary_Data

## References

[btz664-B1] Cock P.J.A. et al (2009) Biopython: freely available Python tools for computational molecular biology and bioinformatics. Bioinformatics, 25, 1422–1423. 19304878 10.1093/bioinformatics/btp163 PMC2682512

[btz664-B2] Guy L. (2017) phyloSkeleton: taxon selection, data retrieval and marker identification for phylogenomics. Bioinformatics, 33, 1230–1232. 28057682 10.1093/bioinformatics/btw824 PMC5408842

[btz664-B3] Hyatt D. et al (2010) Prodigal: prokaryotic gene recognition and translation initiation site identification. BMC Bioinformatics, 11, 119. 20211023 10.1186/1471-2105-11-119 PMC2848648

[btz664-B12] Karsenti E. et al (2011) A holistic approach to marine eco-systems biology. PLOS Biol., 9, e1001177. 22028628 10.1371/journal.pbio.1001177 PMC3196472

[btz664-B4] Lathe W.C. et al (2000) Gene context conservation of a higher order than operons. Trends Biochem. Sci., 25, 474–479. 11050428 10.1016/s0968-0004(00)01663-7

[btz664-B5] Mistry J. et al (2013) Challenges in homology search: hMMER3 and convergent evolution of coiled-coil regions. Nucleic Acids Res., 41, e121. 23598997 10.1093/nar/gkt263 PMC3695513

[btz664-B6] Parks D.H. et al (2015) CheckM: assessing the quality of microbial genomes recovered from isolates, single cells, and metagenomes. Genome Res., 25, 1043–1055. 25977477 10.1101/gr.186072.114 PMC4484387

[btz664-B7] Parks D.H. et al (2017) Recovery of nearly 8, 000 metagenome-assembled genomes substantially expands the tree of life. Nat. Microbiol., 2, 1533. 28894102 10.1038/s41564-017-0012-7

[btz664-B8] Rinke C. et al (2013) Insights into the phylogeny and coding potential of microbial dark matter. Nature, 499, 431–437. 23851394 10.1038/nature12352

[btz664-B9] Rogozin I.B. et al (2002) Connected gene neighborhoods in prokaryotic genomes. Nucleic Acids Res., 30, 2212–2223. 12000841 10.1093/nar/30.10.2212 PMC115289

[btz664-B10] Simão F.A. et al (2015) BUSCO: assessing genome assembly and annotation completeness with single-copy orthologs. Bioinformatics, 31, 3210–3212. 26059717 10.1093/bioinformatics/btv351

[btz664-B11] Tully B.J. et al (2018) The reconstruction of 2, 631 draft metagenome-assembled genomes from the global oceans. Sci. Data, 5, 170203. 29337314 10.1038/sdata.2017.203 PMC5769542

