# Supplementary material for: miComplete: weighted quality evaluation of assembled microbial genomes
Source: Bioinformatics. 2019 Aug 22;36(3):936–7. doi: 10.1093/bioinformatics/btz664 (PMC9883684; doi:10.1093/bioinformatics/btz664)
Supplement: btz664_Supplementary_Data [file bioinformatics_36_3_936_s2.pdf]

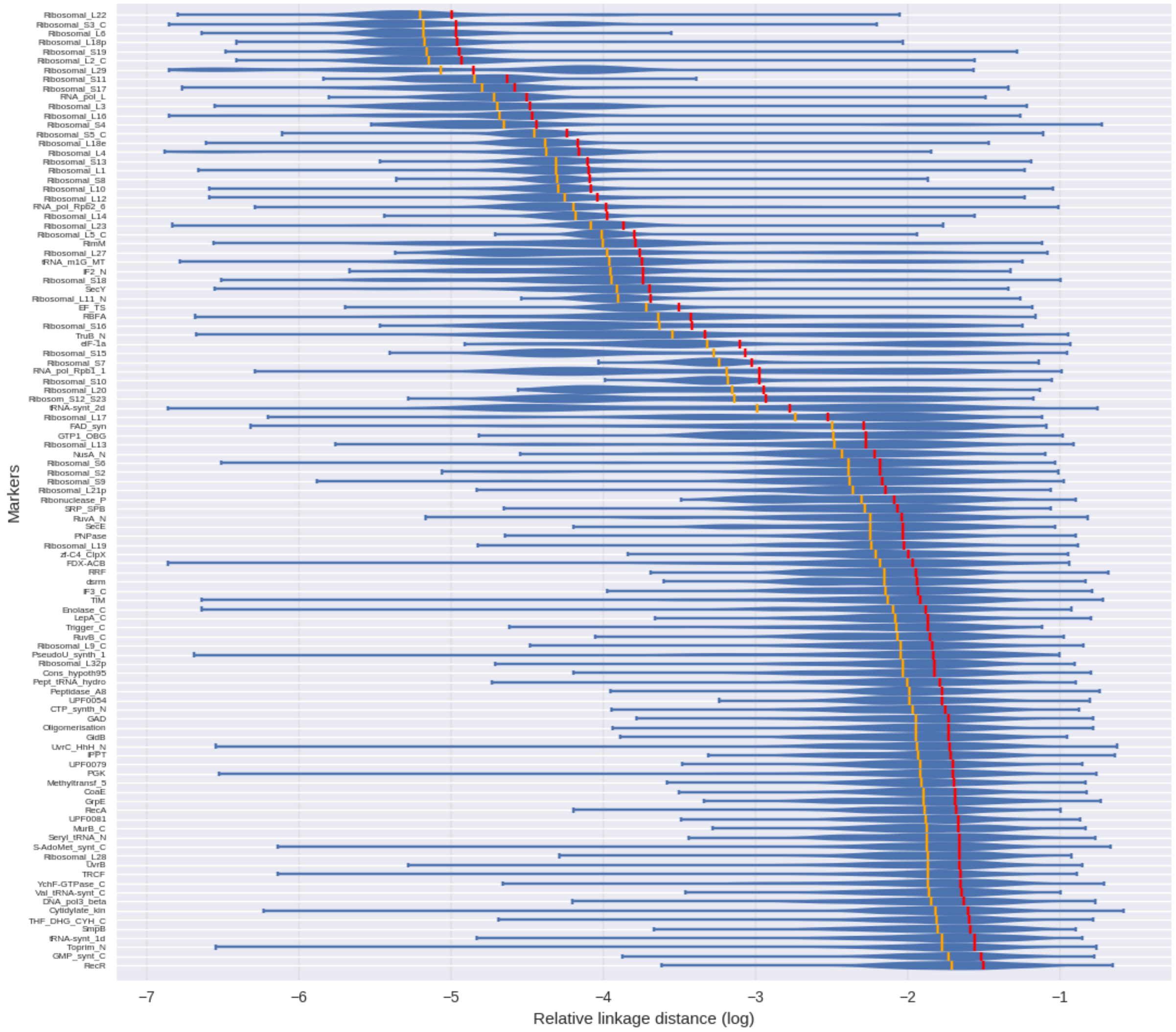

Supplementary Figure 1: Distribution of the relative distances of 105 bacterial markers in a representative set of 1175 genomes. The x-axis represents the $\log_{10}$ value of the relative distance, that is the distance in nucleotides normalized to the chromosome size. A violin plot (blue) is drawn for each marker (y-axis). The orange line represents the median of the distances for each marker, and the red line is the weight, which is the median distance divided by the sum of all median distances, so that the total weight is equal to one. Individual distance for a given marker in a given genome is calculated as the sum of the half the distance to the next marker upstream and half the distance to the next marker downstream.
